# Nutrition in the First 1000 Days: The Origin of Childhood Obesity

**DOI:** 10.3390/ijerph13090838

**Published:** 2016-08-23

**Authors:** Chiara Mameli, Sara Mazzantini, Gian Vincenzo Zuccotti

**Affiliations:** Department of Pediatrics, V. Buzzi Hospital, University of Milan, Milan 20154, Italy; chiara.mameli@unimi.it (C.M.); saramazzantini@hotmail.it (S.M.)

**Keywords:** obesity, overweight, child nutrition, diet, breast feeding

## Abstract

Childhood obesity is a major global issue. Its incidence is constantly increasing, thereby offering a threatening public health perspective. The risk of developing the numerous chronic diseases associated with this condition from very early in life is significant. Although complex and multi-factorial, the pathophysiology of obesity recognizes essential roles of nutritional and metabolic aspects. Particularly, several risk factors identified as possible determinants of later-life obesity act within the first 1000 days of life (i.e., from conception to age 2 years). The purpose of this manuscript is to review those key mechanisms for which a role in predisposing children to obesity is supported by the most recent literature. Throughout the development of the human feeding environment, three different stages have been identified: (1) the prenatal period; (2) breast vs. formula feeding; and (3) complementary diet. A deep understanding of the specific nutritional challenges presented within each phase might foster the development of future preventive strategies.

## 1. Introduction

Despite increased social awareness and numerous preventive public health interventions, childhood obesity is still a major global issue. The most recent epidemiology estimates indicate a 30% prevalence of overweight-to-obese status (Body Mass Index (BMI) > 85th percentile) among children in the United States, steadily increasing over the last twenty years [1]. According to data collected by the World Obesity Federation in 2012, while the Region of Americas consistently shows the highest prevalence, European-based pediatric cohorts also reached alarming rates [2]. As well, several low and middle income developing countries worldwide have reported rapidly rising rates of childhood obesity, despite persisting high levels of undernutrition [3]. We are dealing with a true epidemic, with potentially devastating consequences, particularly considering the health-care-related burden of pediatric-onset obesity being carried along into adult life.

Evidence suggests that several early-life factors significantly contribute to the development of obesity [4]. Particularly, the prenatal and infancy stages can be considered as the key steps in the determination of the individual risk of developing such a condition. The concept of the “first 1000 days” has thereby been described throughout the most recent literature [5,6,7]. The period from conception to 2 years of age is considered the most critical for the induction of those pathophysiological derangements eventually leading up to childhood and then later-life obesity. Any intervention whose aim is to reduce the risk of such imprinting to occur should therefore be focused on this specific early-life period.

It is now clear that obesity is a complex multi-factorial disease with a number of biological triggers, intrinsically entangled with social and environmental influences [8,9]. The purpose of this review is to summarize those factors currently being identified by the literature as the main determinants of pediatric obesity. We will focus on issues related to the nutritional exposure of children during the first 1000 days of life. Within this time frame, three main steps of human dietary development can be identified: (1) the prenatal period; (2) breast vs. formula feeding; and (3) complementary diet. The identification of nutritional risk factors to which children might be exposed during these three phases (Table 1) might be a helpful guide for the development of potential preventive strategies, as well as the background for future research directions.

## 2. Prenatal Period

Important risk factors for childhood obesity can be traced back to maternal influences on the offspring’s metabolism occurring during the gestational period [10,11]. Substantial evidence can be found in the literature on the association between childhood overweight status and both (1) higher maternal pre-pregnancy BMI (pBMI); and (2) excess maternal Gestational Weight Gain (GWG) [12]. To date, these are considered to be the two most important vertically-transmitted obesity risk factors, with an extensive number of publications supporting the associations [4].

In a meta-analysis, Yu et al. pooled data from as many as 45 high-quality eligible studies in order to determine whether pBMI is related to offspring obesity. As a result, the authors showed that, compared with normal-weight controls, pre-pregnancy obese mothers showed a three-fold significantly increased risk for the offspring to develop the disease (Odds Ratio (OR) 3.06, 95% Confidence Interval (CI): 2.68–3.49) [13].

In one of the most recent and influential publications, within the Amsterdam Born Children and their Development (ABCD) cohort study, a large (*n* = 1727) multi-ethnic group of non-diabetic mothers with at term-born children was analyzed. The aim of the study was to determine whether maternal pBMI was associated with offspring adiposity, assessed at age 5–6 years. The authors reported that a significantly increased Odds Ratio for the development of infancy overweight status could be determined for the unit increase in pBMI. The authors also established a link between child adiposity and the maternal blood lipid profile, measured at early gestation (total cholesterol, triglycerides, apolipoproteins A1 and B, and total free fatty acids) [14].

The most recent guidelines by the American College of Obstetricians and Gynecologists indicate a 12-to-16 kg GWG for normal-weight women (BMI: 18.5–24.5 kg·m^−2^), while a significantly lower range is recommended for overweight (BMI: 25–29) and obese (BMI > 30) mothers (7–12 kg and 5–9 kg, respectively) [15].

The earlier work by Fraser et al. examined the association of GWG with offspring adiposity and cardiovascular risk factors at age 9 years, in a large prospective cohort of a UK mother–offspring pairs population. The authors reported that women with greater GWG were more likely to have children with higher BMI, fat mass, systolic blood pressure, C-reactive protein, leptin, and interleukin-6 levels, together with lower high-density lipoprotein cholesterol and apolipoprotein A1.

More recently, Gaillard et al. conducted a prospective cohort study in an Australian population of 3392 mother–offspring pairs in order to investigate the role of pBMI and GWG as potential determinants of increased adolescent (median 17 years) adiposity and cardio-metabolic risk. Maternal weight was obtained pre-pregnancy, at 16.5 ± 2.2 (early pregnancy) and 34.1 ± 1.5 weeks of gestation (late pregnancy). As expected, higher pBMI was associated with higher adolescent BMI, waist circumference, waist-to-hip ratio, systolic blood pressure, insulin, glucose, and insulin-resistance levels. A higher GWG in early-pregnancy, but not mid-pregnancy, was also associated with higher adolescent adiposity. Higher early-pregnancy weight gain corresponded to an increased risk for the adolescent to belong to a high metabolic risk cluster population (OR 1.23, 95% CI: 1.03, 1.47, per Standard Deviation (SD) unit increase in early-pregnancy GWG). The authors report that higher adolescent BMIs, however, inevitably confounded most of the cardio-metabolic risk-assessment analysis [16].

Maternal diabetes mellitus, either gestational or Type 1, also seems to put the offspring at significant risk for developing obesity [17,18,19,20]. However, no specific discussion of the evidence regarding the role of maternal diabetes will be provided in the present review.

The exact connection between obesity and such prenatal metabolic derangements is incompletely understood. It is possible that altered intrauterine conditions can program the fetus to be more prone to obesity due to increased exposure to nutrients being transferred through the placental circulation [21]. However, it must be considered that the epidemiological influence of concurrent lifestyles and socio-environmental features of the overweight-to-obese maternal population might very well enhance the described pathophysiological association [22]. Nevertheless, genetics still plays an important role [23]. A fascinating effort to establish genetic evidence of causal association between maternal BMI (and associated traits) with birth weight is offered by Tyrrell et al. in a very recent original investigation [24]. Applying a Mendelian randomization design, the authors tested whether maternal genetic scores for BMI and obesity-related traits are causally related to the newborn’s weight in a 30,487 women–offspring population. The authors concluded that, in their large cohort, a genetically elevated maternal BMI and blood glucose levels were significantly associated with higher offspring birth weight, thereby genetically exposing children to an increased risk of obesity development [24].

Intriguingly, there is a growing interest around the identification of stable, heritable patterns of altered gene expression due to exogenous exposures leading to the development of obesity [25,26]. Nutritional factors themselves might indeed act through epigenetic modifications to cause long-term programming of obesity risk [27,28]. This might provide us with an explanation of how the human organism seems to long-term perpetuate the effects of such early-life exposures to determine the development of obesity much later in life, possibly further transmitting such susceptibility to the next generation [29]. Targeted metabolomics and epigenomic profiling is currently under way in the European-based Meta-Growth project, aimed at the identification of mechanisms regulating body composition and growth [30].

## 3. Breast vs. Formula Feeding

Moving on to the postnatal early feeding environment, at present, one of the most widely recognized factors at play when discussing the nutritional background of childhood obesity is the known protective role of breastfeeding [31,32].

In 1981, Kramer first postulated the role of breastfeeding in preventing later-life obesity [33]. To date, in Western countries, the concept of the nutritional superiority of breast milk over formula preparation in terms of obesity prevention is well accepted [34,35]. Population studies seem to confirm such observations, even if an exact assessment of risk prevention is made difficult by several confounding factors, thereby leading to an inconsistent body of evidence [36]. Moreover, the literature solely relies on observational studies, being considered unethical to randomize breastfeeding as an intervention. In addition, the significant differences between social and behavioral characteristics of the breast vs. formula feeding populations always bias the analysis [37]. Careful consideration must then be reserved, reading through each publication, to the adopted breastfeeding definitions, particularly in terms of duration and/or exclusivity [38]. A recent meta-analysis of thirty prospective studies was conducted to determine the relative influence of every known potential risk factor for child obesity development. Comparing breastfed with non-breastfed infants, the authors found a 15% decrease in the odds of childhood overweight incidence [39]. However, it must also be acknowledged that most of the available literature in favor of the protective effect of breastmilk towards childhood obesity might not be exactly replicable if applied to different and improved artificial milk formulations developed later on.

From a pathophysiological standpoint, the most widely accepted explanation for the protective effect of breastfeeding is the difference in child growth rates associated with breast vs. formula-fed infants [40,41]. Breastfed infants are known to present a slower growth curve compared to formula-fed children, a difference repeatedly called into question as a protective factor towards later-life obesity [42]. A likely rationale for such a “growth acceleration hypothesis” can be traced back to the higher plasma Insulin-like Growth Factor (IGF)-1 levels shown by formula-fed infants [43]. This might be a potential consequence of the endocrine modulation induced by key differences in bioactive nutrient composition in human vs. formula milk. Particularly, breastmilk is lower in (a) energy and (b) protein, and higher in (c) fat than most commercial formulas [32].

### 3.1. Energy

Increased milk volumes being consumed, and a higher energy density of formula, lead to a 15%–23% higher total energy intake in 3 to 18 month-old formula-fed infants [44]. Patterns of milk intake also vary, with formula-fed infants consuming up to 20%–30% higher volumes per feed. They also tend to eat fewer, larger meals, and to feed less frequently through the night [45]. Moreover, a higher energy intake endures in formula-fed infants when complementary foods are added to the diet. Conversely, breast-fed infants seem to better match energy needs, with breastmilk intake declining once solid foods are added [44,46]. Such a difference in energy intake may exert an obesogenic effect on the formula-fed infant. According to Ong et al., each additional 100 kcal/day consumed at 4 months was associated with 46% higher odds of being overweight at 3 years among a cohort of British children [47].

### 3.2. Protein

Most artificial milk formulations contain a higher protein content, as much as 50%–80% more, compared to breastmilk [40]. This huge discrepancy has been hypothesized to be the main determinant of the growth differences between breast- and formula-fed infants [48]. According to the “early protein hypothesis”, a higher protein intake during formula and complementary feeding significantly influences the child’s growth pattern, thereby increasing the likelihood of obesity development [31]. The numerous publications from the group of Koletzko et al. strongly support this hypothesis [49,50]. The multicenter European Childhood Obesity Program (CHOP) trial was a landmark study in which 1138 healthy, formula-fed infants were randomly assigned to receive, for their first year of life, cow milk-based infant and follow-on formula with either low or high protein content. Six hundred and nineteen exclusively breastfed children were also followed. A significantly increased weight could be assessed in the high-protein group [51].

### 3.3. Fat

Differently from protein, fat content is higher in human milk than in commercially-available formulas [52]. More importantly, breastmilk contains a different concentration of long-chain polyunsaturated fatty acids [53]. To our knowledge, no study has yet to find a significant association between fat intake in infancy and early childhood vs. weight gain or BMI. Higher levels of breast-milk fatty acids are associated with lower glucose levels in the skeletal muscle of breast-fed infants. Reduced plasma levels of pro-inflammatory cytokines can also be assessed [54].

Besides these critical nutrient substrate differences, unlike formula, breast milk composition varies between mothers over the time-course of lactation, and even within each feed. Thus, a close mother-to-offspring nutritional interrelation exists. Through such relationship, the infant’s energetic needs and feeding behaviors—such as frequency and duration of feeds—can be modulated, thereby possibly affecting weight gain [32]. Moreover, breastfed children tend to be at lower risk due to the healthier nutritional environment of their families [34]. As well, a few studies suggest latching to the maternal breast, as opposed to the bottle, led the newborn to develop a more efficient self-regulation of the amount of milk to be consumed [55].

Taken together, these differences in energy and protein balance, feeding patterns, and dietary environment experienced by the formula-fed infant during the first 6 months of age could enhance the risk of developing obesity in later life.

## 4. Complementary Feeding and Early Diet

Finally, our third and last step considers the influence on the risk of obesity incidence due to the nutritional exposure of the child from 6 to 24 months of age. This particular time of the child’s life offers peculiar challenges, mainly due to the transition from breast/formula feeding to complementary early solid diet.

Reviewing the literature, rapid weight gain is the most frequently analyzed risk factor for obesity in this particular period [5]. Even though definitions vary, a significant association is confirmed between higher infancy weight gain and later childhood overweight status [56]. Botton et al. studied anthropometric measures in 468 adolescents aged 8–17 years according to weight and height growth velocities at different ages between birth and 5 years. The authors showed that weight growth velocity at 3 months of age was associated with the development of overweight status in adolescence (OR for a 1-SD increase: 1.52; 95% CI: 1.04, 2.22) [57].

Infant early solid feeding modalities clearly are to be considered. Convincing evidence is available about the benefit of avoiding early timing of the introduction of solid foods [58]. As a part of Project Viva (an important longitudinal study of mother and child nutritional health conducted by Harvard Medical School [59]), Huh et al. examined the association between the timing of solid food introduction during infancy vs. obesity incidence at 3 years in 847 children. The authors report that, among breastfed infants, the timing of solid food introduction was not associated with increased odds of obesity. Conversely, among formula-fed infants, the introduction of solid foods before 4 months of age was associated with a six-fold increase in the odds of diagnosing obesity at age 3 years [60].

A few studies also analyzed the role of infant nutrient intake in later overweight risk [61]. In the Generation R study (a prospective cohort of 3610 Caucasian preschool children), the intake of higher levels of polyunsaturated fat at 14 months of age was associated with a significantly lower risk of preschool overweight (OR: 0.77, 95% CI: 0.62, 0.96 per SD increase) [62]. Regarding protein intake, Scaglioni et al. reported early high protein consumption to be associated with the development of adiposity [63]. On the other hand, Zuccotti et al. recently performed a cross-sectional study to compare the intake of energy, macronutrients, fiber, sodium and iron, and the anthropometric status of 390 children aged 6 to 36 months. Subjects were enrolled across two different geographically distinct Italian cohorts in order to identify potential intake differences and study their impact in a north-to-south gradient. Nutrient intake was evaluated using a very accurate 7-day weighed food record. Anthropometry, energy intake, and macronutrient intake did not differ between the two cohorts. A significant difference in iron and fiber intake was detected, with lower iron and higher fiber been recorded in the northern group. In the studied population, an overall higher intake of proteins, simple carbohydrates, saturated fats and sodium, and a low intake of iron and fiber were also observed, compared to national reference values. However, anthropometry seemed not to be affected in the studied population [64].

A whole new—and only partially explored—frontier adding to the long list of the potential determinants of obesity is the possibility of the role of the infant gut microbiome [65]. Both in animal and human studies, advances in gene sequencing technologies have yielded intriguing evidence in favor of the associations between the gut microbiota and infant weight status [66]. Both milk and early diet shape the differential bacterial colonization of the intestine during development. Emerging evidence suggests that to be a critical pathway linking early feeding environments to later obesity and cardiometabolic risk [67]. The underlying pathophysiology might be associated with the role of intestinal flora in the processing of indigestible polysaccharides into simple sugars and short-chain fatty acids. Since bacteria differ in their energy extracting capabilities, colonization patterns can influence infant growth and, therefore, long-term energy absorption and adipose development [68]. Few data exist to date. However, the composition of the intestinal flora, specifically the relative proportion of *Firmicutes* to *Bacteroides*-type bacteria, seems to be associated with obesity in both animal models and adult human studies [69].

## 5. Conclusions

An extensive body of evidence is already available, and is continuously growing, addressing the relationship between nutrition in early life and the development of the disease later in childhood, as well as in adult age. The first 1000 days, from conception to 24 months, are a temporal window within which some of the most powerful obesity risk factors seem to be identifiable. A clear understanding of the several different mechanisms involved, and their interrelation, is of outstanding importance, particularly for the pediatrician. Maternal influences during gestation, breastfeeding, and early diet implementation all present potential targets for medical and public health interventions aimed at reducing the incidence of childhood obesity.

## Figures and Tables

**Table 1:** Main risk factors in the first 1000 days for the development of childhood obesity.

Nutritional Phase	Risk Factor
**Prenatal (0–280 days)**	Higher maternal pre-pregnancy BMI
	Excess maternal Gestational Weight Gain
	Maternal Diabetes Mellitus (gestational or Type 1)
	Genetic predisposition
**Breast/Formula Feeding (280 days–6 months of age)**	Formula feeding
	● Accelerated growth curve
	● High energy intake
	● High protein content
	● Low concentration of polyunsaturated fatty acids
**Complementary and Early Diet (6 months–2 years of age)**	Rapid weight gain
	Early introduction of solids
	High protein intake
	Gut microbiome
